# Coevolution analyses illuminate the dependencies between amino acid sites in the chaperonin system GroES-L

**DOI:** 10.1186/1471-2148-13-156

**Published:** 2013-07-22

**Authors:** Mario X Ruiz-González, Mario A Fares

**Affiliations:** 1 Integrative and Systems Biology Group, Instituto de Biología Molecular y Celular de Plantas, Consejo Superior de Investigaciones Científicas (CSIC-UPV), Valencia, SPAIN; 2 Department of Genetics, University of Dublin, Trinity College Dublin, Dublin 2, Dublin, Ireland

## Abstract

**Background:**

GroESL is a heat-shock protein ubiquitous in bacteria and eukaryotic organelles. This evolutionarily conserved protein is involved in the folding of a wide variety of other proteins in the cytosol, being essential to the cell. The folding activity proceeds through strong conformational changes mediated by the co-chaperonin GroES and ATP. Functions alternative to folding have been previously described for GroEL in different bacterial groups, supporting enormous functional and structural plasticity for this molecule and the existence of a hidden combinatorial code in the protein sequence enabling such functions. Describing this plasticity can shed light on the functional diversity of GroEL. We hypothesize that different overlapping sets of amino acids coevolve within GroEL, GroES and between both these proteins. Shifts in these coevolutionary relationships may inevitably lead to evolution of alternative functions.

**Results:**

We conducted the first coevolution analyses in an extensive bacterial phylogeny, revealing complex networks of evolutionary dependencies between residues in GroESL. These networks differed among bacterial groups and involved amino acid sites with functional importance and others with previously unsuspected functional potential. Coevolutionary networks formed statistically independent units among bacterial groups and map to structurally continuous regions in the protein, suggesting their functional link. Sites involved in coevolution fell within narrow structural regions, supporting dynamic combinatorial functional links involving similar protein domains. Moreover, coevolving sites within a bacterial group mapped to regions previously identified as involved in folding-unrelated functions, and thus, coevolution may mediate alternative functions.

**Conclusions:**

Our results highlight the evolutionary plasticity of GroEL across the entire bacterial phylogeny. Evidence on the functional importance of coevolving sites illuminates the as yet unappreciated functional diversity of proteins.

## Background

Heat-shock proteins, also known as molecular chaperones, belong to a highly conserved set of protein families that perform essential functions to the cell in prokaryotes and eukaryotes [1]. These functions include, but are not limited to, protein folding, assembly, and transport [2-9]. While the folding function of GroEL has been extensively characterized, emerging literature uncover many alternative functions and structures for this protein (For a recent review see [10]). Mutations in this molecule that are responsible for the emergence of alternative functions remain uncharacterized. Therefore, the potential evolvability of this essential protein is largely unexplored.

GroES and GroEL, also known as cpn10 and cpn60 respectively, are expressed at constitutive levels under physiological conditions and their expression increases at high temperatures, allowing the growth and survival of bacteria at a broad range of temperatures [11-13]. Both chaperonins are encoded by the operon *groE* and they form a homotetradecamer organized into two back-to-back oriented rings. Each of the rings comprises seven identical GroEL subunits, with each subunit being divided into three domains: the apical, which binds unfolded proteins and GroES, the intermediate, which acts as a hinge allowing the movement of the apical domain as well as the transition between *trans* and *cis* conformations needed for GroEL function, and the equatorial which is responsible for the ATPase and the folding activities that take place in the central cavity of the ringed complex [14-16].

The main function of GroEL has been considered to be the folding of other proteins in the cell [6,14,17-20], although evidence supports other folding-unrelated roles for GroEL, such as immune response in humans [21-23] or growth and biofilm formation in bacteria, among others [24-30]. These functions are context dependent and may vary from one organism to another. Alternative functions may emerge in proteins after the duplication and evolution of their encoding gene or through amino acid replacements that impinge on the protein structure. The gene *groEL* has undergone many duplications in bacteria [2], adaptive evolution [31] and functional divergence [32]. Moreover, structural evolutionary changes have been recently described for GroEL, according to which changes in the amino acid composition of its co-chaperonin GroES can determine GroEL functioning as a single instead of double ring [33].

The strong evolutionary sequence conservation of *groEL* and the high number of interactions it establishes with other proteins in the cell [13,34] contrast with GroEL´s functional and structural plasticity and its propensity to persist in duplicate in some bacteria. Particularly striking is the fact that, while performing essential functions in the cell, GroEL presents alternative functions [10]. The trade-off between *groEL*´s high conservation at the sequence and functional levels and its high propensity to evolve novel functions remains poorly understood.

Researchers have attempted to uncover GroEL’s multi-functionality through the testing of the effects of directed mutagenesis of GroEL amino acids under laboratory-controlled conditions. However, the multifunctional nature of GroEL suggests the existence of a reservoir of functionalities resulting from the interaction between distinct sets of amino acids in different bacteria. Here we propose the hypothesis that the functional plasticity of GroEL is mediated by an evolutionary plasticity of potentially functional amino acids. In support of this hypothesis, bacteria growing under different physiological conditions present GroEL variants with functions alternative to folding and which involve different sets of amino acids. The strong selective constraints acting on GroEL imply important functional and structural links between amino acids. These links impose reciprocal selection pressures among amino acid sites. Therefore, changes on GroEL functions from one bacterial group to another should be reflected in strong coevolutionary signatures between linked amino acids whose evolvability is co-regulated by selection in a particular bacterial clade.

In this study we performed an exhaustive coevolutionary analysis using an extensive bacterial phylogeny to uncover the evolutionary, hence functional, dependencies among amino acid residues within GroES, GroEL and between both these proteins. The coevolutionary networks identified in these chaperonins from hundreds of bacteria reveal the complexity underlying the evolution of this essential protein and shed light on the functional importance of previously uncharacterized residues.

## Results

### Sequence data and coevolution analyses

To perform intra-protein coevolution analyses in GroES and GroEL, we searched *groE* sequences amongst the major bacterial Phyla and found that Actinobacteria, Cyanobacteria, Bacteroidetes and Chlorobi, Firmicutes, Proteobacteria, and Spirochaetes comprised a number of *groE* homologs that would allow accurate inference of coevolution. The number of sequences ranged between 11 and 252 for *groES* genes, and 12 and 278 for *groEL* genes belonging to Spirochaetes and Proteobacteria groups, respectively (Table 1). In spite of the differences in the number of sequences, the mean amino acid sequence divergence was of the same order in all bacteria groups ranging between 0.302 and 0.403, and these divergence levels were not correlated with the number of sequences in the alignment. These divergence levels are also within the levels ensuring robust results when using coevolution analyses. Inter-protein coevolution analyses between *groES* and *groEL* were performed building pairs of files for each group of bacteria, both of which included the same bacterial strains. Accordingly, the size of the alignments used for the GroES-L inter-coevolution analyses ranged between 11 in Cyanobacteria and 215 in Proteobacteria (Table 1). All coevolution analyses were performed with a phylogenetic tree built up function in CAPS and pairs of coevolving sites were further filtered through a novel bootstrap analysis (see Methods). Therefore, the number of sequences in the alignment, level of sequence divergence and new introduced filters warranted minimizing false positives rate and increasing accuracy of our results.

**Table 1:** GroES (Cpn10) and GroEL (Cpn60) sequences used in our analysis

**Groups**	**Cpn10**	**Cpn60**	**Cpn10-Cpn60**
Actinobacteria	50	25	18
Aquificae	5	3	-
Bacteroidetes/Chlorobi	29	26	25
Chlamydia/Verrucomicrobia	10	3	-
Chloroflexi	5	4	-
Cyanobacteria	29	13	11
Deinococcus-Thermus	4	4	-
Dictyoglomi	-	1	-
Elusimicrobia	1	1	-
Fibrobacteres/Acidobacteria	3	1	-
Firmicutes	110	118	102
Fusobacteria	1	1	-
Nitrospirae	1	-	-
Proteobacteria (α, β, γ, δ, ϵ)	252	278	215
Proteobacteria Unclassified	1	1	-
Spirochaetes	11	12	10
Tenericutes	1	8	-
Thermotogae	5	6	-
Unclassified	1	-	-
All groups	519	505	381

For the individual intra-group analyses we chose those bacterial groups with more than 10 sequences. For the overall Cpn10 and Cpn60 intra-group analyses we took all sequences (519 and 505 respectively).

### Evolutionary dependencies between functional sites within GroES and GroEL

To determine the magnitude of the evolutionary plasticity of GroEL and GroES, we first conducted a coevolutionary analysis to determine the network of residues dependencies in all bacteria. We performed intra-protein coevolution analyses in a 519 sequences based GroES alignment and 505 sequences based GroEL alignment, representing the 6 major bacterial groups. We also calculated the support of each pair of coevolutionary sites taking into account the phylogenetic relationships using a non-parametric bootstrap approach (see Material and Methods for details). All amino acid sites numbering and composition are referred throughout the text to the numbering in the crystal structure of GroESL from *E. coli* (1AON.pdb).

**Figure 1:**
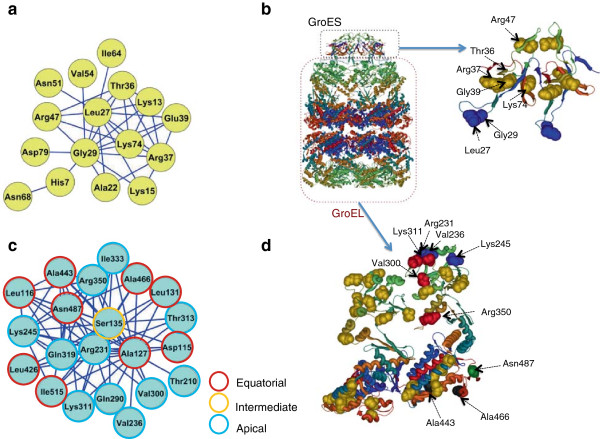
**Coevolution analyses within GroES and GroEL.** The network of coevolving amino acid sites within GroES is shown using the three-letter amino acid code **(a)** Sites coevolving within GroES were divided into two main structure clusters **(b)** One cluster includes two amino acid sites (blue spheres), which are involved in the interaction with GroEL. The second cluster includes residues (yellow spheres) mapping to the inter-GroES subunit faces. The network of coevolution in GroEL **(c)** identifies amino acid sites which are involved in the interaction with GroES and protein substrates **(**blue spheres in the structure of GroEL: **d)** sites involved in the inter-subunit GroEL contacts and and substrate folding in the ring cavity (red spheres), residues with a role in ATP hydrolysis (green sphere) and those mapping to the inter-ring interfaces (black spheres).

We identified a single connected network of 16 coevolving amino acid sites in GroES, with Lys13, Leu27, Gly29, Thr36, Arg37, Glu39, Arg47 and Lys74 establishing most of the evolutionary dependencies (Figure 1a). To determine the importance of each of the amino acid sites in the network (e.g., amino acids establishing most of the connections) we applied network centrality measures to coevolving sites, typically used in networks biology: degree centrality, betweenness and closeness. Networks are a collection of points joined together in pairs by lines. In the networks jargon, points are referred to as vertices or nodes while the links are referred to as edges. Centrality measures of nodes, including degree, betweenness and closeness, are typically used to determine the importance of these nodes in the network. Degree is the number of edges departing from a node in the network. A node presents high closeness when its shortest distances to all other nodes in the network are low compared to the average closeness. A node has high betweenness when the number of shortest paths between all pairs of nodes in a network that pass through it is high.

Interestingly, Leu27 and Gly29, two amino acids known to be involved in the interaction between GroES and GroEL [35,36] are the most central in the coevolution network (Additional file 1: Figure S1a to c). The dependency of these two essential amino acids on other functionally uncharacterized ones hints possible functional links between both sets of amino acid sites. Indeed, Lys13, Thr36, Arg37, Gly39, Arg47 and Lys74, while lacking apparent functions, they form a structural cluster establishing important contacts among GroES subunits (Figure 1b). Amino acid sites within each of the structural clusters were in close proximity to each other (for example, their proximal carbon atoms were less than 4 Å distant, against an average distance of 40 Å between all pairs of amino acids). Coevolution among structurally proximal amino acid sites is a general pattern [37] and suggests compensatory relationships, hence functional or structural links, between amino acids [38-40].

In GroEL, we identified 21 coevolving amino acid residues (Figure 1c), of which Leu116, Ala127, Ser135, Arg231, Lys245, Gln319, Arg350, Ala443, and Asn487 were the most central residues to the network (Additional file 1: Figure S1d to 1f). Arg231, Val236, and Lys245 are involved or close to (less than 4 Å distance in the structure) sites mediating substrate and GroES binding. Other positions were either included or close to charged amino acid sites that were facing the central GroEL cavity (for example, Gln290, Val300, Lys311, and Arg350). Finally, Asn487 is located in the ATP and Mg^2+^ binding site, while other amino acid sites, such as Ala443 and Ala466, are at the rings interface and likely involved in protein folding within the GroES-L ring complex. All 21 amino acids are distributed into two structural groups: one in the apical and another in the equatorial domains (Figure 1d). Remarkably, coevolving sites are very close to sites involved in protein folding, substrate and GroES binding, ATP binding and hydrolysis, or inter-subunits contacts, thus, suggesting that changes at these amino acids may have important functional consequences (Figure 1d).

### Coevolution of GroES with GroEL

The interaction of GroES and GroEL is essential to induce the conformational changes needed for the folding cycle. These conformational changes may force coadaptation dynamics between GroES and GroEL.

**Figure 2:**
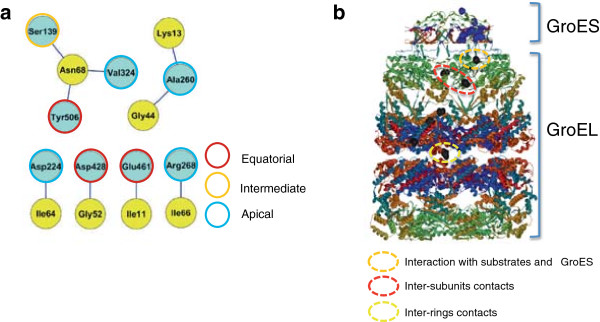
**Coevolution between GroES and GroEL.** The network of residues involved in the evolutionary dependency between GroES and GroEL identifies 7 residues from GroES and 8 from GroEL **(a)** Structural mapping of coevolving residues reveals the functional importance of coevolving residues **(b)** residues coevolving between both proteins belong to substrate binding regions, inter-subunit and inter-ring contacts.

We performed coevolutionary analyses using the protein sequences of GroES and GroEL from the same set of bacterial strains (381 sequences for GroES and GroEL). These sequences span all the different bacterial groups (Table 1), with all these groups being well represented. Analysis of coevolution identified a group of amino acids from GroES coevolving with GroEL (Figure 2a). The centrality measures of coevolving sites were also calculated (Additional file 2: Figure S2a to c). Coevolution did not affect GroES sites involved in the GroES-L interaction. Nonetheless, sites coevolving between both proteins had important functional roles and mapped to different functional domains of GroEL. For example, two of the GroEL sites, Ala260 and Arg268, are involved in the binding of substrates and overlap with sites involved in GroES binding as well [35]. In addition, Glu461, involved in the coevolution between Ala260 and Arg268, has a role in stabilizing inter-ring contacts [41]. Since GroES is heavily involved in determining the function of GroEL as a single or as a double ring [33], the coevolution of Glu461 from GroEL with GroES amino acid sites may have implications in the structural stability of the double ring, and thus, GroES-GroEL folding cycle.

In support of the structural and functional communication between the coevolving sites of GroES and GroEL, coevolving amino acids formed structural clusters within GroESL (Figure 2b). In addition to their clustering, coevolving sites were either functionally relevant or were close to sites with reported functional importance. Taken together, these results support the hypothesis that the coevolutionary relationships are the result of selective constraints on amino acid sites that are structurally or functionally linked in the GroES-L complex.

### Shifts of GroES-GroEL coevolutionary relationships during bacterial evolution

We tested whether the coevolutionary relationships among amino acid sites have changed among the different bacterial groups, which would indicate functional changes in GroES-L. Functional shifts in GroEL have been previously documented and linked to events of GroEL gene duplication [32] and to changes in the organismal lifestyle [10,32]. However, a precise analysis of the sites potentially driving GroEL functional changes in major bacterial groups has not been conducted before.

We identified evolutionary dependencies between amino acid sites that were specific to a particular bacterial group but not to others. Previous studies have shown that the number of sequences in the alignment may undermine the accuracy of coevolution-detection methods [42]. To avoid such size-dependent effects, we performed bootstrap analyses of the coevolving pairs of sites (see material and methods). Amino acid sites identified as coevolving presented high bootstrap values (Additional file 3: Figure S3 and Additional file 4: Figure S4 for the coevolution results of GroES and GroEL, respectively). Amino acid sites detected in coevolution analyses between GroES and GroEL (Additional file 5: Figure S5) were not detected in intra-protein coevolution analyses, and thus, were not the result of indirect evolutionary dependencies.

**Figure 3:**
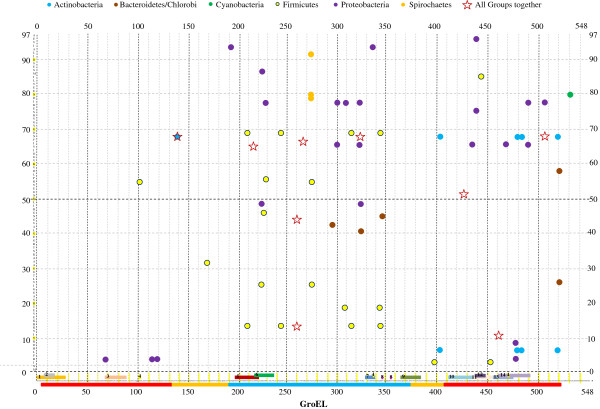
**Identifying shifts in coevolutionary links between amino acids in the different bacterial clades.** We have analysed the coevolution of GroES and GroEL in the different bacterial clades (colour coded circles) and compared involved residues with those identified across the entire bacterial phylogeny (stars). The distribution of the coevolving residues along GroEL is shown in the X-axis, while this distribution in GroES is shown in the Y-axis. The continuous bar at the very bottom of the figure represents the three different major domains of GroEL (Apical: blue, Intermediate: yellow and Equatorial: red). On top of the continuous bar we have also identified regions reported to be involved in folding-independet functions. These regions are color-coded as in [10]: 1, 3 and 11, orange: binding to mouse adipocytes; 2 and 12, binding to potato leafroll virus; 4, insecticidal neurotoxin; 5, Monocytes and T-cell activators; 6, Binding to primary mouse macrophages; 7 and 9, binding to lipopolysaccharides; 8, insecticidal toxin; 10 and 13, binding to cell surface of J774A.1 cells; 14, monocytes modulation activity.

Amino acid sites from GroEL coevolving with sites from GroES were centred in the apical and equatorial domains (Figure 3). While this was the general pattern when analysing the full alignment, this distribution varied significantly between bacterial clades. Figure 3 represents the distribution of coevolving sites in GroES and GroEL for each of the bacterial groups examined in this study. A brief inspection of the graph allows identifying the sharp differences in the distribution of sites in the different domains of GroEL. For example, in Firmicutes coevolving sites (yellow filled circles) concentrated mainly in the apical domain, in good agreement with the distribution of such sites when analysing the entire set of bacteria (red stars). Proteobacteria (purple filled circles) presented one set of coevolving sites in the apical domain and another in the C-terminal equatorial domain. Finally, in Actinobacteria (blue filled circles) all but one coevolving site were located in the C-terminal domain of GroEL.

**Figure 4:**
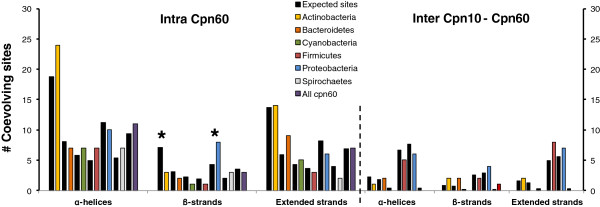
**Distribution of coevolving sites amongst secondary structures in GroEL.** The observed number of sites within each structure (colour coded bars according to the bacterial group) was compared to the expected number of such sites using a χ^*2*^ distribution. Significant values (*P* < 0.05) are indicated with black stars.

The distribution of coevolving sites in GroEL secondary structures and domains also differed among bacterial groups. Figure 4 represents the distribution of the expected number and the number of coevolving sites observed in Figure 3 in the alpha helices, beta-strands and extended strands. The main differences in the distribution of coevolving sites among bacterial groups reside in the Beta-strands. Beta-strands were significantly enriched for sites under coevolution in Proteobacteria, non-enriched in other bacterial groups, and significantly impoverished in Actinobacteria. These data are in good agreement with the functional and structural differences in GroEL found between Proteobacteria and Actinobacteria [10].

Coevolving sites are three-dimensionally proximal in the structure of GroES and GroEL. For example, His7 and Asn68 from Actinobacteria that are strongly proximal in the structure (mean Euclidean distance between their proximal atoms is less than 4 Å) were coevolving with two sets of amino acids from GroEL. One set included Tyr478, Ala481 and Cys519, all three being very proximal to one another in the equatorial domain of GroEL, and another set comprised Cys138 and His401, which were proximal in the intermediate domain.

To determine the functional meaning of the groupings of coevolving sites in each bacterial clade, we performed two different analyses. First, we followed a previously published approach to define functional sectors in GroEL and GroES [43]. In this study, sectors are characterized by statistical independence, structural continuity, biochemical independence and divergence independence. Halabi and colleagues [43] showed that statistical protein sectors correspond to functional sectors. We tested three of the sectors properties using computational means: statistical and divergence independences and structural continuity. Second, we mapped sites identified as coevolving in one bacterial group but not in other into those protein regions known to have shifted GroEL function to other folding unrelated functions in that bacterial group.

### Groups of coevolution form protein sectors statistically independent among bacteria

Functional links between sites impose correlation in their entropies [43]. To test this, we measured the amount of conservation (*D*_*i*_) for the sites of each GroEL protein domain as a function of Entropy (see Material and Methods for details). Then, we calculated the correlation entropy (*I*_*i*_) for each group of coevolving sites (see Material and methods). To determine if the group of coevolving sites within a bacterial clade is independent from that of another bacterial clade, we compared the correlation entropy of groups of different bacterial clades for each of the GroEL domains. Three were the domains compared (apical, equatorial and intermediate domains) between bacterial groups. If the change in the sites composition of coevolution networks is the result of functional shifts between bacteria, sites within a network in a bacterial group (*g1*) should correlate in their entropies (*I*_*i*_) more than with any of the sites of the network of the other bacterial group (*g2*). That is, the entropy correlation of one group should be independent of that of the other group (*I*_*g1-g2*_*≈ I*_*g1*_*+I*_*g2*_).

A main difference between our approach and that of the previous study [43] is that sectors in our approach are defined based on coevolution analyses derived from CAPS, while those of Halabi and colleagues [43] were identified using statistical coupling analyses (SCA) to determine the contribution of correlations to conservation profiles.

**Figure 5:**
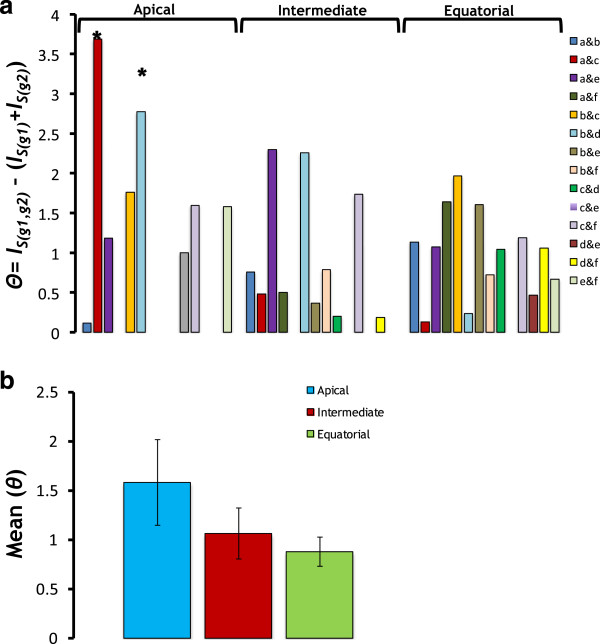
**Groups of coevolving sites correlate in their entropies forming independent protein sectors.** We measured entropy and correlation entropies for each pair of groups belonging to different bacteria using the equations 1 to 4 from the text. Compared groups were taken from the same protein domain (Apical, Intermediate or Equatorial). Bacteria groups compared included Actinobacteria **(a)** Bacteroidetes **(b)** Cyanobacteria **(c)** Spirochaetes **(d)** Firmicutes **(e)** and Proteobacteria **(f)**. Two groups of coevolution (*g1* and *g2*) were considered independent when the joined correlation entropy for the groups (*I*_*S(g1,g2)*_) was approximately equal to the sum of correlation entropies (*I*_*S(g1)*_) and (*I*_*S(g2)*_). The significance of the difference between these two parameters [Θ = *I*_*S(g1,g2)*_ – (*I*_*S(g1)*_ + *I*_*S(g2)*_)] was tested against a null distribution of Θ drawn from a 1000 groups built by randomly sampling sites from the same protein domain. Significant Θ values under a normal test (*P* < 0.05) are indicated with *.

Analyses of correlation entropies showed that all groups of coevolving sites within the apical domain for a bacterial group were independent from those in other bacterial groups (Figure 5a) (e.g., comparison of *θ* = *I*_*g1-g2*_*–* (*I*_*g1*_*+I*_*g2*_*)* from the real group with a set of 1000 pseudorandom replicates yield no significant difference between the two groups (*g1* and *g2*)). The same was inferred for the groups of coevolving sites from the intermediate domain of GroEL. Conversely, in the apical domain we found independent groups of coevolution for all bacterial groups with the exception of Spirochaetes, in which *I*_*g1-g2*_ was much smaller than (*I*_*g1*_*+I*_*g2*_*)* (Figure 5a). Comparison of the mean differences (*θ*) indicates that equatorial domain showed the strongest signal of functional sectors independence among bacterial strains, followed by the intermediate and apical domains (Figure 5b). These differences were not, however, statistically significant under a Wilcoxon ranked test.

### Groups of coevolution present structural continuity

**Figure 6:**
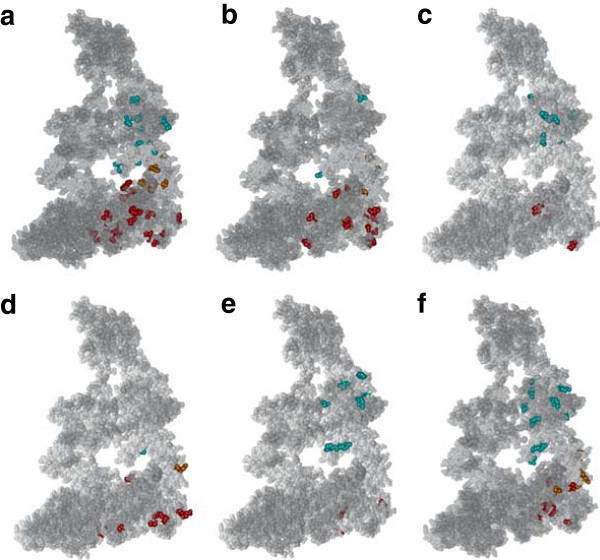
**Distribution of groups of coevolving sites within the three domains of crystal structure of GroEL (1AON.pdb).** We compared these distributions using a dimer GroEL_2_-GroES_2_. The groups of bacteria represented are Actinobacteria **(a)** Bacteroidetes **(b)** Cyanobacteria **(c)** Spirochaetes **(d)** Firmicutes **(e)** and Proteobacteria **(f)**. Sites under coevolution are highlighted as solid spheres, with those belonging to the same group colour-coded. Sites falling within the apical, intermediate and equatorial domains are coded with the colours blue, yellow and red, respectively.

To determine if the sites within a coevolution group were linked structurally within a bacterial clade, we plotted them into the crystal structure of *E. coli* GroESL proteins complex. Figure 6 presents evidence of the structural clustering of sites within each of the bacterial groups in the three protein domains. Importantly, the coevolutionary shifts between bacterial groups are apparent and their structural mapping provides insights into the possible functional differences among the groups of coevolving residues. A remarkable observation is that amino acids that coevolved in one group of bacteria are located in a completely different structure face to those detected in another group of bacteria, while both keeping structural continuity. As a case in point, the alpha helices populated with coevolving amino acids in Proteobacteria are independent from those in Actinobacteria. This rule applies to both, the equatorial and the apical domains (Figure 6a and f). In addition to the difference in structural patterns, Proteobacteria present coevolving amino acids in regions involved in protein folding while Actinobacteria are mostly affected in the surfaces of subunits mediating the inter-ring contacts. This differential distribution supports functional shifts between both bacterial clades, with one having larger effect on folding while the other on the stability of the GroEL double ring complex. Another striking example of functional and structural differentiation is that of Spirochaetes, with most of the coevolving amino acids mapping to the inter-ring regions of the equatorial domain (Figure 6d).

### Coevolution of GroEL sites with folding-independent functions

GroEL regions responsible for functional differences among bacteria are reported in Figure 4 of [10]. We have compared the sites coevolving in one bacterial clade but not another and plotted these sites in the different domains known to confer GroEL alternative non-folding functions. Many of the sites involved in a coevolutionary relationship in a bacterial group have been reported to be involved in a GroEL function alternative to protein folding (Figure 3). For example, two of the coevolving sites in Actinobacteria are directly involved in monocyte modulation by the Actinobacterium *Micobacterium tuberculosis* ([44], figure 3). Moreover, a number of the amino acids identified as coevolving exclusively in proteobacteria map to a region from GroEL previously found to bind to potato leafroll virus and to facilitate its movement in the plant [45,46] (Figure 3). The extensive list of coevolving amino acid sites mapping within these folding-alternative functions (Figure 3) is testament to the important implications of groups of coevolution in the functional plasticity of GroEL.

## Discussion

### Complex coevolutionary networks in GroESL define the functional boundaries of amino acid sites

Our analyses of the coevolutionary dynamics within GroES and GroEL as well as between both these interacting proteins uncover a complex network of evolutionary dependencies among amino acid sites. These dependencies often involve sets of sites with known functional relevance but also comprise other sites with unknown importance. However, the functional importance of these untested sites is supported by a number of observations and tests made in this study. First, we show that most amino acids involved in coevolutionary dynamics are three-dimensionally clustered in the protein structure and closely located to functionally or structurally important sites. As a case in point, functionally important sites in GroES present the largest centrality values in GroES coevolutionary network, indicating their greater evolutionary dependencies with other sites closely located in the protein structure. The coevolution of sites surrounding important functional regions may compensate the effects of mutations at these functional sites or near functional and catalytic pockets, thereby maintaining an overall volume or shape for that pocket [37]. Our results on the proximity of coevolving sites to functional domains support previous studies claiming that covarying groups of amino acid sites are often identified at critical protein regions [37,40,47-52]. Second, covarying amino acid sites identified in this study are part of networks that correspond to structural clusters, that is, these sites fall close to each other in the protein structure. In conclusion, the low number of sites identified in our coevolutionary analyses, their structural clustering, and their proximity to functional or proteins interface regions point to their functional or structural importance. This is supported by previous studies indicating that sites coevolving with few others within the protein are likely to represent functional dependencies [49,53,54].

Most covarying amino acid sites in GroEL were identified in the equatorial and apical domains and only few sites were located in the intermediate domain. Apical and equatorial domains perform most functions in GroEL. It is remarkable that many of the amino acids from the equatorial domain involved in coevolutionary relationships belong to the most carboxi-terminal GroEL tail. Indeed, the folding of substrates within the central GroEL cavity is favoured by the limited size and hydrophobicity of the cavity [6,20]. The C-terminal tail of GroEL define the environment within the central cavity of GroEL with regards to its hydrophobicity, which would impact on both the size and nature of the substrate proteins folded by the chaperonin [55]. Collectively, our results uncover a list of amino acid sites that might have profound implications on the functions of GroES and GroEL.

### The evolutionary dependencies between GroES and GroEL provide information on the structural consequences of their interaction

Our coevolutionary analyses in GroES and GroEL identified several sets of sites with apparently distinct roles. First, GroES amino acid regions coevolving with residues from GroEL are all located in the interface between the GroES subunits. Second, GroEL residues coevolving with GroES are distributed among the three domains, apical, intermediate and equatorial. In the apical domain, two amino acid residues coevolving with GroES are involved in substrate binding. One site is located at the interface between the two GroEL heptameric rings and may be involved in the stabilization of these domains. Indeed, the folding reaction cycle requires the double ring of GroEL, in which the information passes between the rings to signal the ATP hydrolysis progress in one ring and which causes important conformational changes in the opposite ring [56,57]. One such change involves the weakening of GroES-GroEL binding, which ends with the binding of an ATP to the opposite ring [58]. The inter-ring amino acid contacts are, therefore, essential for the folding cycle completion and release of GroES from the *cis* ring once ATP has been bound to the opposite ring. Arguably, coevolution between the interface of the rings and GroES may be the result of the constraints to maintain the structural communication between the two GroEL rings upon the interaction with GroES.

### Amino acids coevolution underlies the functional plasticity of GroES and GroEL in bacteria

Our results bring forward the controversial, although intuitive, suggestion that the function of a protein may change across an evolutionary scale leading to a plastic fitness landscape in which constraints on amino acids can vary dramatically. Against the static view of one protein one function, we propose that proteins have the potential to perform many alternative functions. Leaping from one function to another requires the correlated evolution of key amino acids in the protein. GroEL, and its co-chaperonin GroES, offer a unique system to test this hypothesis because, despite its essentiality to the cell, this protein has evolved many alternative functions in other bacteria [21-30]. The performance of alternative functions is dependent on the fixation of mutations in genes. Since amino acids are constrained by their interactions with other amino acids, fixation of mutations at sites with functional relevance must be accompanied by mutations in other sites of the protein through molecular coadaptation dynamics—that is, amino acids that are structurally or functionally linked exercise reciprocal natural selection on one another [59].

The groups of amino acids identified in the intra-protein and inter-protein coevolution analyses differed between bacterial groups, in good agreement with the apparent difference in functions of GroEL in these bacteria. Groups of coevolving amino acids in one domain of a bacterial group showed statistical and structural independence of that in the same domain from another bacterial group. Many of the coevolution groups found in one bacterial group map to regions of *groEL* that are known to encode functions alternative to protein folding. Other coevolving amino acids could not be directly mapped to domains with known alternative functions, though their structural proximity to these domains hints potential roles for these sites. Remarkably, the set of amino acid sites involved in an evolutionary dependency in one bacterial group was close in the protein structure to the set of amino acids detected for another bacterial group. In fact, in some cases, the same amino acid was detected as coevolving with different sets of amino acids in two bacterial groups, thereby acting as evolutionary hinges of alternative functional protein sectors. For example, in the intra-GroEL coevolution analysis, Met514 was detected in Actinobacteria and Bacteroidetes, but it was coevolving with different amino acids in these two groups. The general trend was that alternative sets of coevolving sites identified in different bacteria were closely located in the structure. This supports the plausible hypothesis that shifts in the selective constraints on amino acid sites of GroEL are subtle between bacteria, and affect the same structural regions; probably those regions undergoing conformational changes when GroEL interacts with GroES.

To conclude, we provide evidence of the plasticity of the evolutionary relationships between the amino acid sites in an essential protein. We also list a set of coevolving sites that might be worth testing for addressing important questions regarding the functional promiscuity of GroEL and its evolvability under different conditions. Experimental studies aimed at determining the importance of the amino acid sites listed in this study may aid the development of mechanistic models of protein folding in the cell and the evolution of alternative functions from highly conserved ones.

## Conclusions

Our results map genetic diversity in GroESL to its functional promiscuity. While different functional sectors in GroESL can be assigned to distinct functions, the overlap in the amino acids sets of these sectors put forward the conclusion that functional leaps in proteins can be driven by subtle sequence compositional differences. Our results highlight the evolutionary plasticity of GroEL across the entire bacterial phylogeny. Evidence on the functional importance of coevolving sites illuminates the as yet unappreciated functional diversity of proteins.

## Methods

### Sequences, alignments and phylogenetic inference

All GroES and GroEL (also known as cpn10 and cpn60, respectively) sequences where downloaded from the OMA browser site (http://omabrowser.org). We used either cpn10 or cpn60 and *Rhizobium* as keywords. Then we chose the link to the page with the highest number of orthologs, RHIL300891 (Q1MKX3), with 903 orthologs (01/04/2011) for cpn10 and RHIL300890 (CH601_RHIL3), with 870 orthologs (23/03/2011). We removed all eukaryotic and archaeal sequences prior to the analysis. Then, we aligned all sequences using ClustalX2 [60,61]. The output alignment was manually refined using GeneDoc 2.6 [62] and this new alignment was used to build a neighbor-joining tree with 1000 bootstrap replicates in ClustalX2. The trees were visualized with FigTree 1.3.1 (http://tree.bio.ed.ac.uk/software/figtree/) and all redundant sequences (same amino acidic sequences) were detected and deleted but leaving a representative one. Then, we removed the sequences belonging to duplicated genes within all given species, ending with a final alignment that included 519 sequences for the cpn10 and 505 sequences for the cpn60 (see Table 1). We used CAPS [50] to analyse the intra-protein coevolution clustering of amino acids for both the cpn10 and cpn60 alignments. For both alignments we used a threshold *α* value of 0.001, a random sampling of 100000, and a bootstrap value of 100. In addition to these two alignments, we prepared new alignments for those taxonomic groups with at least 10 sequences for both cpn10 and cpn60 proteins (sample sizes in Table 1): Actinobacteria, Bacteroidetes/Chlorobi group, Cyanobacteria, Firmicutes, all Proteobacteria together, and Spirochaetes. In these analyses the bootstrap values were adapted to the sample sizes (20, 80, 100, 20, 10, and 9, respectively).

To conduct coevolution analysis between GroES and GroEL, we built multiple sequence alignments for both of the proteins, which comprised the sequences belonging to the same organismal source (a total of 381 sequences for GroES and GroEL, Table 1). We downloaded the sequences for the crystallized cpn10 and cpn60 proteins of *Escherichia coli* (PDB ID: 1AON, MMDB ID: 47936) from the NCBI site (http://www.ncbi.nlm.nih.gov/sites/structure) to map the coevolving amino acidic sites detected using CAPS in the protein structure. Since the output amino acidic sites detected by CAPS correspond to the position in the input alignment, which included gaps, we wrote a script in C++ (Microsoft Visual C++ Standard Edition 6.0, available from authors upon request) to identify the coevolving sites in the sequence of the published structure of the protein. The networks of coevolving amino acids were performed using Cytoscape 2.8.2 [63]. The crystal structure of GroESL complex was represented using the software imol (P. Rotkiewicz, http://www.pirx.com/iMol/index.shtml).

### Coevolution analyses

Coevolution analyses, that is the correlated variation of two amino acid sites throughout the multiple sequence alignment, was performed using a previously published coevolution method [64] implemented in the program CAPS [50]. Other Mutual Information methods were used as well but their performance was significantly poorer, providing large sets of sites and false positive results in agreement with a previous study [64]. Briefly, this method estimates how correlated is the evolutionary variability at two sites of the same or different protein-coding multiple sequence alignments. To account for the strength of the amino acids transitions in a site, the BLOSUM score of amino acid transitions of a site between two sequences was corrected by the time since the divergence of the two sequences compared. Time of divergence was calculated using the Li’s corrected synonymous nucleotide substitutions. Phylogenetic artifacts—phylogeny asymmetries, long-branch attractions, and unequal codon and base composition biases among the bacterial clades—were accounted for by conducting the same coevolution analyses in a set of neutrally evolving simulated alignments, which bear the same evolutionary features as the real sequence alignments. A pair of sites was considered to coevolve if the probability of their correlation coefficient was lower than 0.001 when compared to the null distribution of such coefficients drawn from the simulated sequence alignments. Moreover, to identify coevolving pairs of sites that may be functionally or structurally linked across the bacterial phylogeny, we conducted non-parametric bootstrap analyses of covariation (see next section).

### Bootstrapping the pairs of coevolving sites

In this study, we have devised a new method to determine the reliability of a coevolution pair of amino acid sites. This test is based upon the assumption that pairs of sites involved in important functional roles within a phylogenetic group should be inextricably linked between each other with regards to their evolutionary patterns, such that the two sites of the pair should be evolutionarily dependent on one another through their reciprocal natural selection. That is, a change in one amino acid should be accompanied by a compensatory (coadaptive) change in its coevolving amino acid partner. Making the inverse rationale, pairs of amino acid sites that are consistently detected as coevolving in a phylogenetic context should be functionally related.

For each of the pairs of amino acid sites detected in our coevolutionary analyses, we performed a non-parametric bootstrapping, that is we randomly sampled sequences from the phylogenetic tree, performed the coevolutionary analyses for those sampled sequences using CAPS and, then, checked whether a particular pair of sites detected in the real coevolutionary analyses was also detected in this new sampled dataset. We replicated this procedure a 1000 times and, then, asked how many times each of the pairs of sites detected as coevolving in the real multiple sequence alignments was detected as significantly supporting coevolution. Those pairs that were identified in more than 70% of the phylogenetic random samples were deemed as consistently coevolving amino acid sites.

### Measuring statistical independence of coevolutionary groups among bacteria

To measure the statistical independence of group of coevolving sites from another, we first calculated the entropy of the group (D_S_):

(1) $$D_{S}=f_{i,j,\ldots ,S}^{\left(a\right)}\ln\frac{f_{i,j,\ldots ,S}^{\left(a\right)}}{q^{\left(a\right)}}+\left(1-f_{i,j,\ldots ,S}^{\left(a\right)}\right)\ln\frac{1-f_{i,j,\ldots ,S}^{\left(a\right)}}{1-q^{\left(a\right)}}$$

Here $f_{i,j,\ldots ,S}^{\left(a\right)}$ is the frequency of the most represented amino acid (a) in each of the sites under coevolution (*i*, *j*, …, *S*) within the group. This frequency is compared to the frequency of the amino acid (*a*) in all the proteins (*q*^*(a)*^).

Then, we measured the correlation entropy of the group (*I*_*S*_) as:

(2) $$I_{S}=D_{S}-\underset{i\in S}{\sum }D_{i}^{\left(a\right)}$$

where, $D_{i}^{\left(a\right)}$ is the frequency of the amino acid (*a*) at site *i* and is calculated as:

(3) $$D_{i}^{\left(a\right)}=f_{i}^{\left(a\right)}\ln\frac{f_{i}^{\left(a\right)}}{q^{\left(a\right)}}+\left(1-f_{i}^{\left(a\right)}\right)\ln\frac{1-f_{i}^{\left(a\right)}}{1-q^{\left(a\right)}}$$

Two groups (*g*_*1*_ and *g*_*2*_) are independent of one another, if their correlation entropies follows:

(4) $$I_{S\left(g_{1},g_{2}\right)}\approx I_{S\left(g_{1}\right)}+I_{S\left(g_{2}\right)}$$

To determine the significance of the difference between both sides of equation 4, we built 1000 groups, each with the same size as the coevolution group; then, we estimated *I*_*S(g1)*_ and *I*_*S(g2)*_, and compared this to *I*_*S(g1,g2)*_.

## Competing interests

The authors declare that they have no competing interests.

## Authors’ contributions

MAF devised and designed the study. MXRG prepared all the multiple sequence alignments for this study. MAF and MXRG conducted the analyses of coevolution. MAF performed the statistical analyses and wrote the final version of this manuscript. Both authors read and approved the final manuscript.

## Supplementary Material

Additional file 1: Figure S1 Importance of amino acid sites in the coevolutionary netoworks of GroES (a to c) and GroEL (d to f). We used centrality measures to determine how many coevolution links did each of the amino acid sites detected using CAPS have with the other sites in the protein. We used three main centrality measures, including Betweenness, closeness and degree for the networks of GroES (a to c) and GroEL (d to f). Respectively. In these networks, amino acid sites are represented using the three-letter amino acid codes followed by the position of the amino acid in the three-dimensional structure of the GroESL protein complex (PDB ID: 1AON, MMDB ID: 47936). The diameter of the circles is proportional to the centrality of that amino acid site in the network. Click here for file

Additional file 2: Figure S2 Network of coevolution among amino acid sites between GroES and GroEL. The coevolution network between GroES and GroEL (a) is represented by inter-connected circles, each of which contains the three-leter code of the amino acid and the position in the crystal structure of GroESL (PDB ID: 1AON, MMDB ID: 47936). Amino acids belonging to GroES are in yellow circles while those of GroEL are in blue circles. Centrality measures of this network, including Betweenness (b), closeness (c) and degree (d) are also represented. Click here for file

Additional file 3: Figure S3 Network of coevolution among amino acid sites in GroES in different bacterial groups. To identify shifts in the coevolution networks, we analyzed coevolution in GroES in the different bacterial groups and identified amino acid sites with evolutionary dependencies in three groups: coevolution network in Actinobacteria (a); Firmicutes (b) and Proteobacteria (c). We used the numbering of sites according to the structure of GroEL from *Escherichia coli* (PDB ID: 1AON, MMDB ID: 47936). Click here for file

Additional file 4: Figure S4 Network of coevolution among amino acid sites in GroEL in different bacterial groups. We identified coevolution between GroEL residues in six bacterial groups, including Actinobacteria (a), Bacteroidetes (b), Cyanobacteria (c), Spirochaetes (d), Firmicutes (e) and Proteobacteria (f). We used amino acid numberings according to the position of the site in the crystal structure of GroEL from *Escherichia coli* (PDB ID: 1AON, MMDB ID: 47936). The position of the sites in the three domains of GroEL, equatorial, apical and intermediate, is color-coded. Click here for file

Additional file 5: Figure S5 Network of coevolution among amino acid sites between GroES and GroEL in different bacterial groups. We identified coevolution between GroES and GroEL residues in six bacterial groups, including Actinobacteria (a), Bacteroidetes (b), Cyanobacteria (c), Spirochaetes (d), Firmicutes (e) and Proteobacteria (f). We used amino acid numberings according to the position of the site in the crystal structure of GroEL from *Escherichia coli* (PDB ID: 1AON, MMDB ID: 47936). The position of the sites in the three domains of GroEL, equatorial, apical and intermediate, is color-coded. GroES residues are labeled in yellow. Click here for file

## References

[B1] Sakamoto M Ohkuma M Usefulness of the hsp60 gene for the identification and classification of Gram-negative anaerobic rods J Med Microbiol 2010 59 Pt 11 1293 1302 20671088 10.1099/jmm.0.020420-0

[B2] Lund PA Multiple chaperonins in bacteria–why so many? FEMS Microbiol Rev 2009 33 4 785 800 10.1111/j.1574-6976.2009.00178.x 19416363

[B3] Lund PA Microbial molecular chaperones Adv Microb Physiol 2001 44 93 140 11407116 10.1016/s0065-2911(01)44012-4

[B4] Ranson NA White HE Saibil HR Chaperonins Biochem J 1998 333 Pt 2 233 242 9657960 10.1042/bj3330233 PMC1219577

[B5] Radford SE GroEL: More than Just a folding cage Cell 2006 125 5 831 833 10.1016/j.cell.2006.05.021 16751091

[B6] Lin Z Rye HS GroEL-mediated protein folding: making the impossible, possible Crit Rev Biochem Mol Biol 2006 41 4 211 239 10.1080/10409230600760382 16849107 PMC3783267

[B7] Fenton WA Horwich AL GroEL-mediated protein folding Protein Sci 1997 6 4 743 760 9098884 10.1002/pro.5560060401 PMC2144759

[B8] Hayer-Hartl MK Weber F Hartl FU Mechanism of chaperonin action: GroES binding and release can drive GroEL-mediated protein folding in the absence of ATP hydrolysis EMBO J 1996 15 22 6111 6121 8947033 PMC452432

[B9] Mayhew M Da Silva AC Martin J Erdjument-Bromage H Tempst P Hartl FU Protein folding in the central cavity of the GroEL-GroES chaperonin complex Nature 1996 379 6564 420 426 10.1038/379420a0 8559246

[B10] Henderson B Fares MA Lund PA Chaperonin 60: a paradoxical, evolutionarily conserved protein family with multiple moonlighting functions Biol Rev Camb Philos Soc 2013 10.1111/brv.12037 23551966

[B11] VanBogelen RA Acton MA Neidhardt FC Induction of the heat shock regulon does not produce thermotolerance in Escherichia coli Genes Dev 1987 1 6 525 531 10.1101/gad.1.6.525 3315852

[B12] Fayet O Ziegelhoffer T Georgopoulos C The groES and groEL heat shock gene products of Escherichia coli are essential for bacterial growth at all temperatures J Bacteriol 1989 171 3 1379 1385 2563997 10.1128/jb.171.3.1379-1385.1989 PMC209756

[B13] Kerner MJ Naylor DJ Ishihama Y Maier T Chang HC Stines AP Georgopoulos C Frishman D Hayer-Hartl M Mann M Proteome-wide analysis of chaperonin-dependent protein folding in Escherichia coli Cell 2005 122 2 209 220 10.1016/j.cell.2005.05.028 16051146

[B14] Braig K Otwinowski Z Hegde R Boisvert DC Joachimiak A Horwich AL Sigler PB The crystal structure of the bacterial chaperonin GroEL at 2.8 A Nature 1994 371 6498 578 586 10.1038/371578a0 7935790

[B15] Hunt JF Weaver AJ Landry SJ Gierasch L Deisenhofer J The crystal structure of the GroES co-chaperonin at 2.8 A resolution Nature 1996 379 6560 37 45 10.1038/379037a0 8538739

[B16] Xu Z Horwich AL Sigler PB The crystal structure of the asymmetric GroEL-GroES-(ADP)7 chaperonin complex Nature 1997 388 6644 741 750 10.1038/41944 9285585

[B17] Thirumalai D Lorimer GH Chaperonin-mediated protein folding Annu Rev Biophys Biomol Struct 2001 30 245 269 10.1146/annurev.biophys.30.1.245 11340060

[B18] Ellis RJ Chaperomics: in vivo GroEL function defined Curr Biol 2005 15 17 R661 663 10.1016/j.cub.2005.08.025 16139196

[B19] Ellis RJ Protein misassembly: macromolecular crowding and molecular chaperones Adv Exp Med Biol 2007 594 1 13 10.1007/978-0-387-39975-1_1 17205670

[B20] Horwich AL Fenton WA Chapman E Farr GW Two families of chaperonin: physiology and mechanism Annu Rev Cell Dev Biol 2007 23 115 145 10.1146/annurev.cellbio.23.090506.123555 17489689

[B21] Tuccinardi D Fioriti E Manfrini S D’Amico E Pozzilli P DiaPep277 peptide therapy in the context of other immune intervention trials in type 1 diabetes Expert Opin Biol Ther 2011 11 9 1233 1240 10.1517/14712598.2011.599319 21751937

[B22] Zonneveld-Huijssoon E Roord ST De Jager W Klein M Albani S Anderton SM Kuis W Van Wijk F Prakken BJ Bystander suppression of experimental arthritis by nasal administration of a heat shock protein peptide Ann Rheum Dis 2011 70 12 2199 2206 10.1136/ard.2010.136994 21914624

[B23] Ronaghy A De Jager W Zonneveld-Huijssoon E Klein MR Van Wijk F Rijkers GT Kuis W Wulffraat NM Prakken BJ Vaccination leads to an aberrant FOXP3 T-cell response in non-remitting juvenile idiopathic arthritis Ann Rheum Dis 2011 70 11 2037 2043 10.1136/ard.2010.145151 21859687

[B24] George R Kelly SM Price NC Erbse A Fisher M Lund PA Three GroEL homologues from Rhizobium leguminosarum have distinct in vitro properties Biochem Biophys Res Commun 2004 324 2 822 828 10.1016/j.bbrc.2004.09.140 15474501

[B25] Rodriguez-Quinones F Maguire M Wallington EJ Gould PS Yerko V Downie JA Lund PA Two of the three groEL homologues in Rhizobium leguminosarum are dispensable for normal growth Arch Microbiol 2005 183 4 253 265 10.1007/s00203-005-0768-7 15830189

[B26] Ojha A Anand M Bhatt A Kremer L Jacobs WR Jr Hatfull GF GroEL1: a dedicated chaperone involved in mycolic acid biosynthesis during biofilm formation in mycobacteria Cell 2005 123 5 861 873 10.1016/j.cell.2005.09.012 16325580

[B27] Bittner AN Foltz A Oke V Only one of five groEL genes is required for viability and successful symbiosis in Sinorhizobium meliloti J Bacteriol 2007 189 5 1884 1889 10.1128/JB.01542-06 17158666 PMC1855696

[B28] Gould PS Burgar HR Lund PA Homologous cpn60 genes in Rhizobium leguminosarum are not functionally equivalent Cell Stress Chaperones 2007 12 2 123 131 10.1379/CSC-227R.1 17688191 PMC1949324

[B29] Li J Wang Y Zhang CY Zhang WY Jiang DM Wu ZH Liu H Li YZ Myxococcus xanthus viability depends on groEL supplied by either of two genes, but the paralogs have different functions during heat shock, predation, and development J Bacteriol 2010 192 7 1875 1881 10.1128/JB.01458-09 20139189 PMC2838048

[B30] Wang Y Zhang W-Y Zhang Z Li J Li Z-F Tan Z-G Zhang T-T Wu Z-H Liu H Li Y-Z Mechanisms involved in the functional divergence of duplicated GroEL chaperonins in Myxococcus xanthus DK1622 PLoS Genet 2013 9 2 e1003306 10.1371/journal.pgen.1003306 23437010 PMC3578752

[B31] Fares MA Barrio E Sabater-Munoz B Moya A The evolution of the heat-shock protein GroEL from Buchnera, the primary endosymbiont of aphids, is governed by positive selection Mol Biol Evol 2002 19 7 1162 1170 10.1093/oxfordjournals.molbev.a004174 12082135

[B32] McNally D Fares MA In silico identification of functional divergence between the multiple groEL gene paralogs in Chlamydiae BMC Evol Biol 2007 7 81 10.1186/1471-2148-7-81 17519003 PMC1892554

[B33] Liu H Kovacs E Lund PA Characterisation of mutations in GroES that allow GroEL to function as a single ring FEBS Lett 2009 583 14 2365 2371 10.1016/j.febslet.2009.06.027 19545569

[B34] Fujiwara K Ishihama Y Nakahigashi K Soga T Taguchi H A systematic survey of in vivo obligate chaperonin-dependent substrates EMBO J 2010 29 9 1552 1564 10.1038/emboj.2010.52 20360681 PMC3212837

[B35] Buckle AM Zahn R Fersht AR A structural model for GroEL-polypeptide recognition Proc Natl Acad Sci USA 1997 94 8 3571 3575 10.1073/pnas.94.8.3571 9108017 PMC20480

[B36] Fenton WA Kashi Y Furtak K Horwich AL Residues in chaperonin GroEL required for polypeptide binding and release Nature 1994 371 6498 614 619 10.1038/371614a0 7935796

[B37] Gloor GB Martin LC Wahl LM Dunn SD Mutual information in protein multiple sequence alignments reveals two classes of coevolving positions Biochemistry 2005 44 19 7156 7165 10.1021/bi050293e 15882054

[B38] Davis BH Poon AF Whitlock MC Compensatory mutations are repeatable and clustered within proteins Proc Biol Sci 2009 276 1663 1823 1827 10.1098/rspb.2008.1846 19324785 PMC2674493

[B39] Fares MA Computational and Statistical methods to explore the various dimensions of protein evolution Current Bioinformatics 2006 1 207 217 10.2174/157489306777011950

[B40] Codoner FM Fares MA Why should we care about molecular coevolution? Evol Bioinform Online 2008 4 29 38 19204805 PMC2614197

[B41] Brocchieri L Karlin S Conservation among HSP60 sequences in relation to structure, function, and evolution Protein Sci 2000 9 3 476 486 10752609 10.1110/ps.9.3.476 PMC2144576

[B42] Codoner FM O’Dea S Fares MA Reducing the false positive rate in the non-parametric analysis of molecular coevolution BMC Evol Biol 2008 8 106 10.1186/1471-2148-8-106 18402697 PMC2362121

[B43] Halabi N Rivoire O Leibler S Ranganathan R Protein sectors: evolutionary units of three-dimensional structure Cell 2009 138 4 774 786 10.1016/j.cell.2009.07.038 19703402 PMC3210731

[B44] Hu Y Henderson B Lund PA Tormay P Ahmed MT Gurcha SS Besra GS Coates AR A Mycobacterium tuberculosis mutant lacking the groEL homologue cpn60.1 is viable but fails to induce an inflammatory response in animal models of infection Infect Immun 2008 76 4 1535 1546 10.1128/IAI.01078-07 18227175 PMC2292875

[B45] Hogenhout SA van der Wilk F Verbeek M Goldbach RW van den Heuvel JF Potato leafroll virus binds to the equatorial domain of the aphid endosymbiotic GroEL homolog J Virol 1998 72 1 358 365 9420234 10.1128/jvi.72.1.358-365.1998 PMC109383

[B46] Hogenhout SA van der Wilk F Verbeek M Goldbach RW van den Heuvel JF Identifying the determinants in the equatorial domain of Buchnera GroEL implicated in binding Potato leafroll virus J Virol 2000 74 10 4541 4548 10.1128/JVI.74.10.4541-4548.2000 10775590 PMC111974

[B47] Buck MJ Atchley WR Networks of coevolving sites in structural and functional domains of serpin proteins Mol Biol Evol 2005 22 7 1627 1634 10.1093/molbev/msi157 15858204

[B48] Gloor GB Tyagi G Abrassart DM Kingston AJ Fernandes AD Dunn SD Brandl CJ Functionally compensating coevolving positions are neither homoplasic nor conserved in clades Mol Biol Evol 2010 27 5 1181 1191 10.1093/molbev/msq004 20065119

[B49] Tillier ER Charlebois RL The human protein coevolution network Genome Res 2009 19 10 1861 1871 10.1101/gr.092452.109 19696150 PMC2765286

[B50] Fares MA McNally D CAPS: coevolution analysis using protein sequences Bioinformatics 2006 22 22 2821 2822 10.1093/bioinformatics/btl493 17005535

[B51] Travers SA Fares MA Functional coevolutionary networks of the Hsp70-Hop-Hsp90 system revealed through computational analyses Mol Biol Evol 2007 24 4 1032 1044 10.1093/molbev/msm022 17267421

[B52] Travers SA Tully DC McCormack GP Fares MA A study of the coevolutionary patterns operating within the env gene of the HIV-1 group M subtypes Mol Biol Evol 2007 24 12 2787 2801 10.1093/molbev/msm213 17921487

[B53] Tillier ER Lui TW Using multiple interdependency to separate functional from phylogenetic correlations in protein alignments Bioinformatics 2003 19 6 750 755 10.1093/bioinformatics/btg072 12691987

[B54] Little DY Chen L Identification of coevolving residues and coevolution potentials emphasizing structure, bond formation and catalytic coordination in protein evolution PLoS One 2009 4 3 e4762 10.1371/journal.pone.0004762 19274093 PMC2651771

[B55] Tang YC Chang HC Roeben A Wischnewski D Wischnewski N Kerner MJ Hartl FU Hayer-Hartl M Structural features of the GroEL-GroES nano-cage required for rapid folding of encapsulated protein Cell 2006 125 5 903 914 10.1016/j.cell.2006.04.027 16751100

[B56] Yifrach O Horovitz A Nested cooperativity in the ATPase activity of the oligomeric chaperonin GroEL Biochemistry 1995 34 16 5303 5308 10.1021/bi00016a001 7727391

[B57] Horovitz A Fridmann Y Kafri G Yifrach O Review: allostery in chaperonins J Struct Biol 2001 135 2 104 114 10.1006/jsbi.2001.4377 11580260

[B58] Weissman JS Hohl CM Kovalenko O Kashi Y Chen S Braig K Saibil HR Fenton WA Horwich AL Mechanism of GroEL action: productive release of polypeptide from a sequestered position under GroES Cell 1995 83 4 577 587 10.1016/0092-8674(95)90098-5 7585961

[B59] Fares MA Ruiz-Gonzalez MX Labrador JP Protein coadaptation and the design of novel approaches to identify protein-protein interactions IUBMB Life 2011 63 4 264 271 10.1002/iub.455 21488148

[B60] Larkin MA Blackshields G Brown NP Chenna R McGettigan PA McWilliam H Valentin F Wallace IM Wilm A Lopez R Clustal W and Clustal X version 2.0 Bioinformatics 2007 23 21 2947 2948 10.1093/bioinformatics/btm404 17846036

[B61] Thompson JD Gibson TJ Plewniak F Jeanmougin F Higgins DG The CLUSTAL_X windows interface: flexible strategies for multiple sequence alignment aided by quality analysis tools Nucleic Acids Res 1997 25 24 4876 4882 10.1093/nar/25.24.4876 9396791 PMC147148

[B62] GeneDoc Analysis and visualization of Genetic Variation http://www.psc.edu/biomed/Genedoc/

[B63] Smoot ME Ono K Ruscheinski J Wang PL Ideker T Cytoscape 2.8: new features for data integration and network visualization Bioinformatics 2011 27 3 431 432 10.1093/bioinformatics/btq675 21149340 PMC3031041

[B64] Fares MA Travers SA A novel method for detecting intramolecular coevolution: adding a further dimension to selective constraints analyses Genetics 2006 173 1 9 23 10.1534/genetics.105.053249 16547113 PMC1461439

