# Supplementary figures and images for: Coevolution analyses illuminate the dependencies between amino acid sites in the chaperonin system GroES-L
Source: BMC Evol Biol. 2013 Jul 22;13:156. doi: 10.1186/1471-2148-13-156 (PMC3728108; doi:10.1186/1471-2148-13-156)

**a**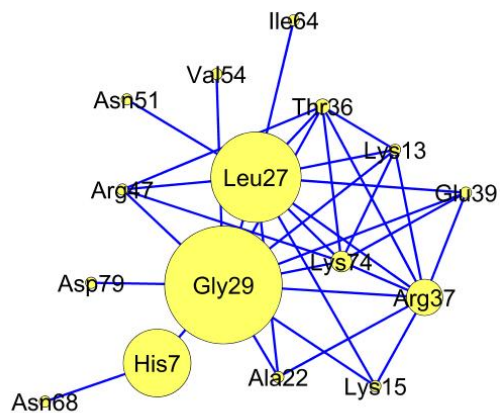**b**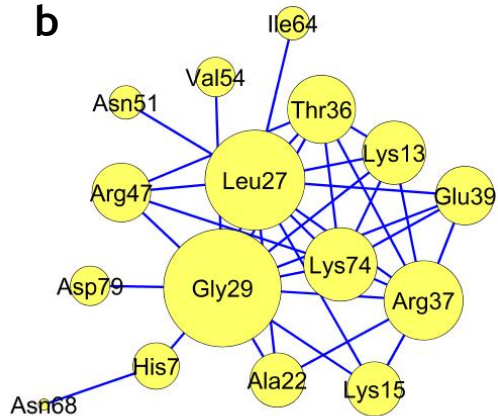**c**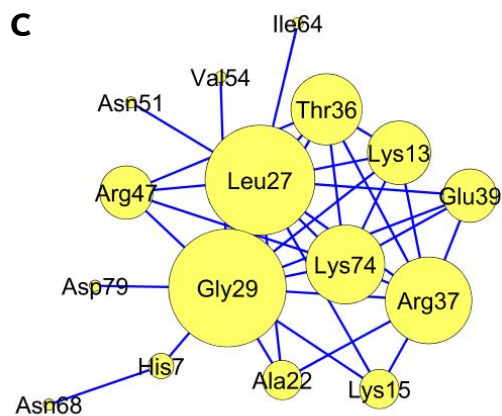**d**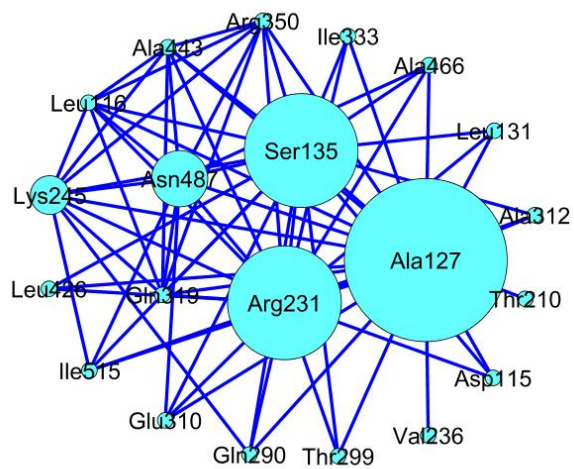**e**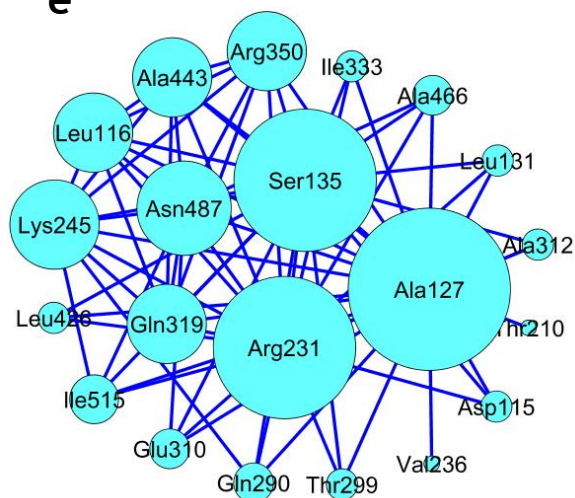**f**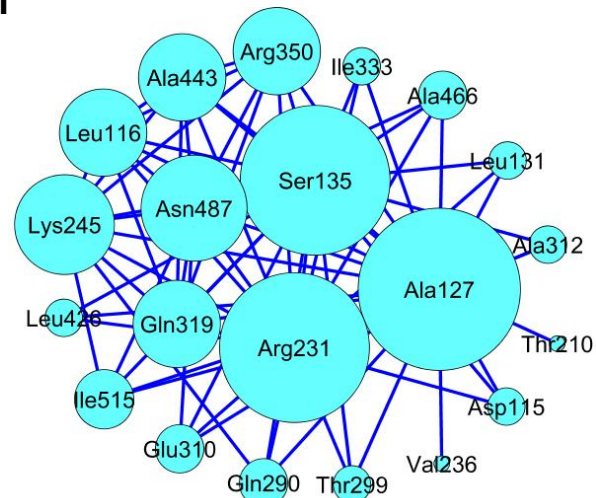

Supplement: Additional file 1: Figure S1 — Importance of amino acid sites in the coevolutionary netoworks of GroES (a to c) and GroEL (d to f). We used centrality measures to determine how many coevolution links did each of the amino acid sites detected using CAPS have with the other sites in the protein. We used three main centrality measures, including Betweenness, closeness and degree for the networks of GroES (a to c) and GroEL (d to f). Respectively. In these networks, amino acid sites are represented using the three-letter amino acid codes followed by the position of the amino acid in the three-dimensional structure of the GroESL protein complex (PDB ID: 1AON, MMDB ID: 47936). The diameter of the circles is proportional to the centrality of that amino acid site in the network. [file 1471-2148-13-156-S1.pdf]

**a**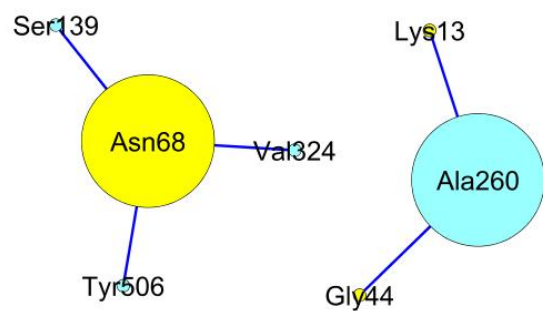**b**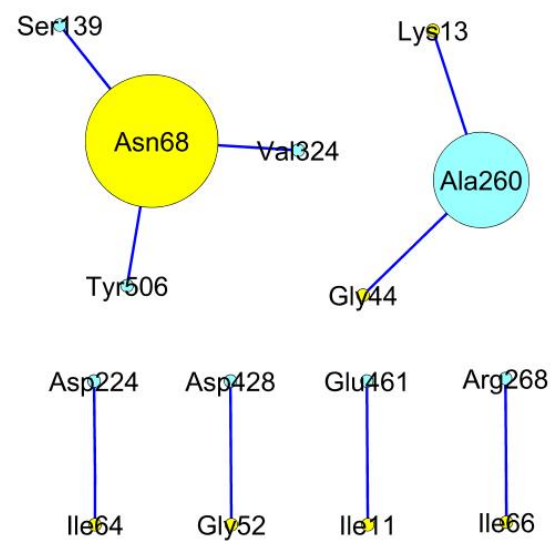**c**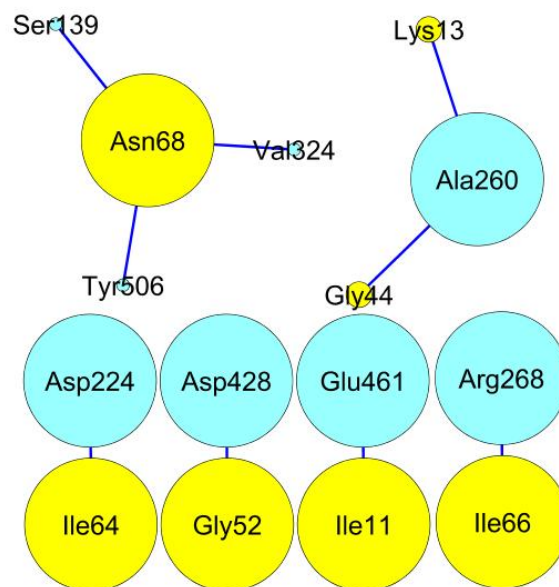

Supplement: Additional file 2: Figure S2 — Network of coevolution among amino acid sites between GroES and GroEL. The coevolution network between GroES and GroEL (a) is represented by inter-connected circles, each of which contains the three-leter code of the amino acid and the position in the crystal structure of GroESL (PDB ID: 1AON, MMDB ID: 47936). Amino acids belonging to GroES are in yellow circles while those of GroEL are in blue circles. Centrality measures of this network, including Betweenness (b), closeness (c) and degree (d) are also represented. [file 1471-2148-13-156-S2.pdf]

**a**

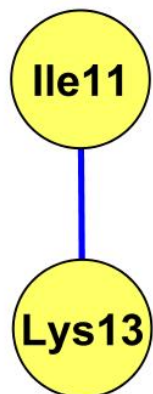

**b**

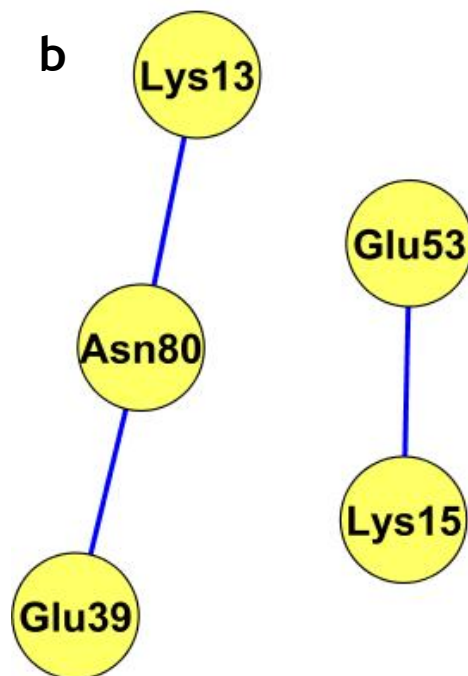

**c**

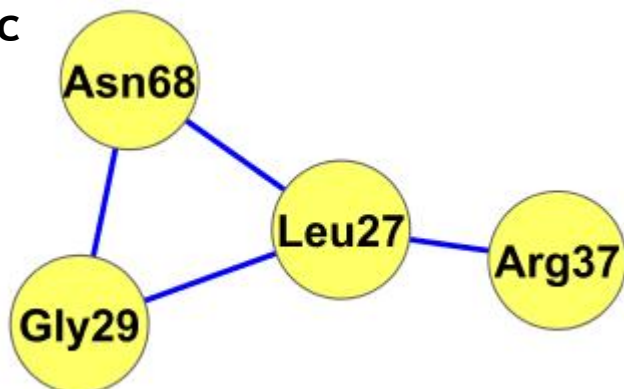

Supplement: Additional file 3: Figure S3 — Network of coevolution among amino acid sites in GroES in different bacterial groups. To identify shifts in the coevolution networks, we analyzed coevolution in GroES in the different bacterial groups and identified amino acid sites with evolutionary dependencies in three groups: coevolution network in Actinobacteria (a); Firmicutes (b) and Proteobacteria (c). We used the numbering of sites according to the structure of GroEL from Escherichia coli (PDB ID: 1AON, MMDB ID: 47936). [file 1471-2148-13-156-S3.pdf]

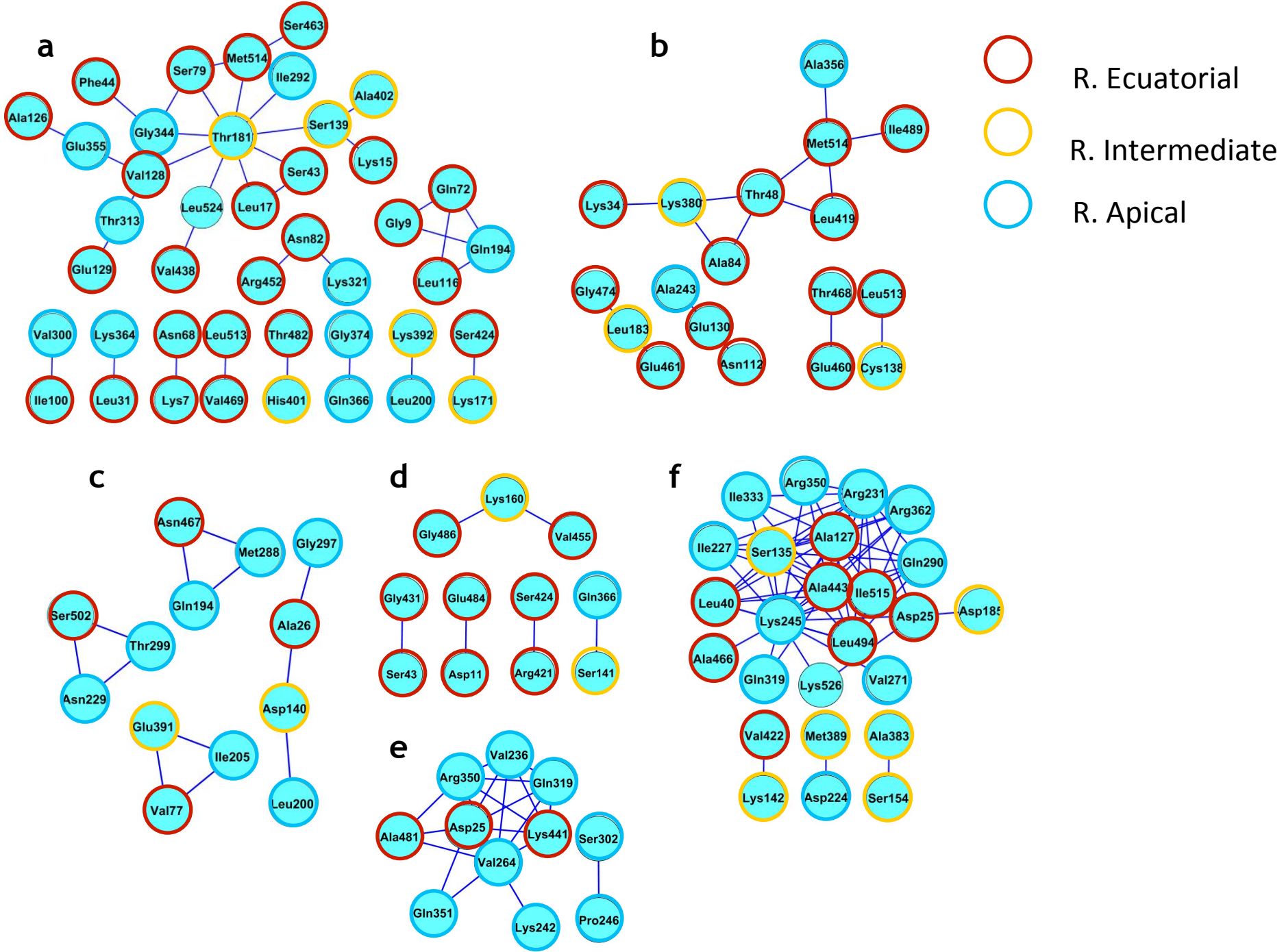

Supplement: Additional file 4: Figure S4 — Network of coevolution among amino acid sites in GroEL in different bacterial groups. We identified coevolution between GroEL residues in six bacterial groups, including Actinobacteria (a), Bacteroidetes (b), Cyanobacteria (c), Spirochaetes (d), Firmicutes (e) and Proteobacteria (f). We used amino acid numberings according to the position of the site in the crystal structure of GroEL from Escherichia coli (PDB ID: 1AON, MMDB ID: 47936). The position of the sites in the three domains of GroEL, equatorial, apical and intermediate, is color-coded. [file 1471-2148-13-156-S4.pdf]
