# Supplementary material for: Coevolution analyses illuminate the dependencies between amino acid sites in the chaperonin system GroES-L
Source: BMC Evol Biol. 2013 Jul 22;13:156. doi: 10.1186/1471-2148-13-156 (PMC3728108; doi:10.1186/1471-2148-13-156)
Supplement: Additional file 5: Figure S5 — Network of coevolution among amino acid sites between GroES and GroEL in different bacterial groups. We identified coevolution between GroES and GroEL residues in six bacterial groups, including Actinobacteria (a), Bacteroidetes (b), Cyanobacteria (c), Spirochaetes (d), Firmicutes (e) and Proteobacteria (f). We used amino acid numberings according to the position of the site in the crystal structure of GroEL from Escherichia coli (PDB ID: 1AON, MMDB ID: 47936). The position of the sites in the three domains of GroEL, equatorial, apical and intermediate, is color-coded. GroES residues are labeled in yellow. [file 1471-2148-13-156-S5.pdf]

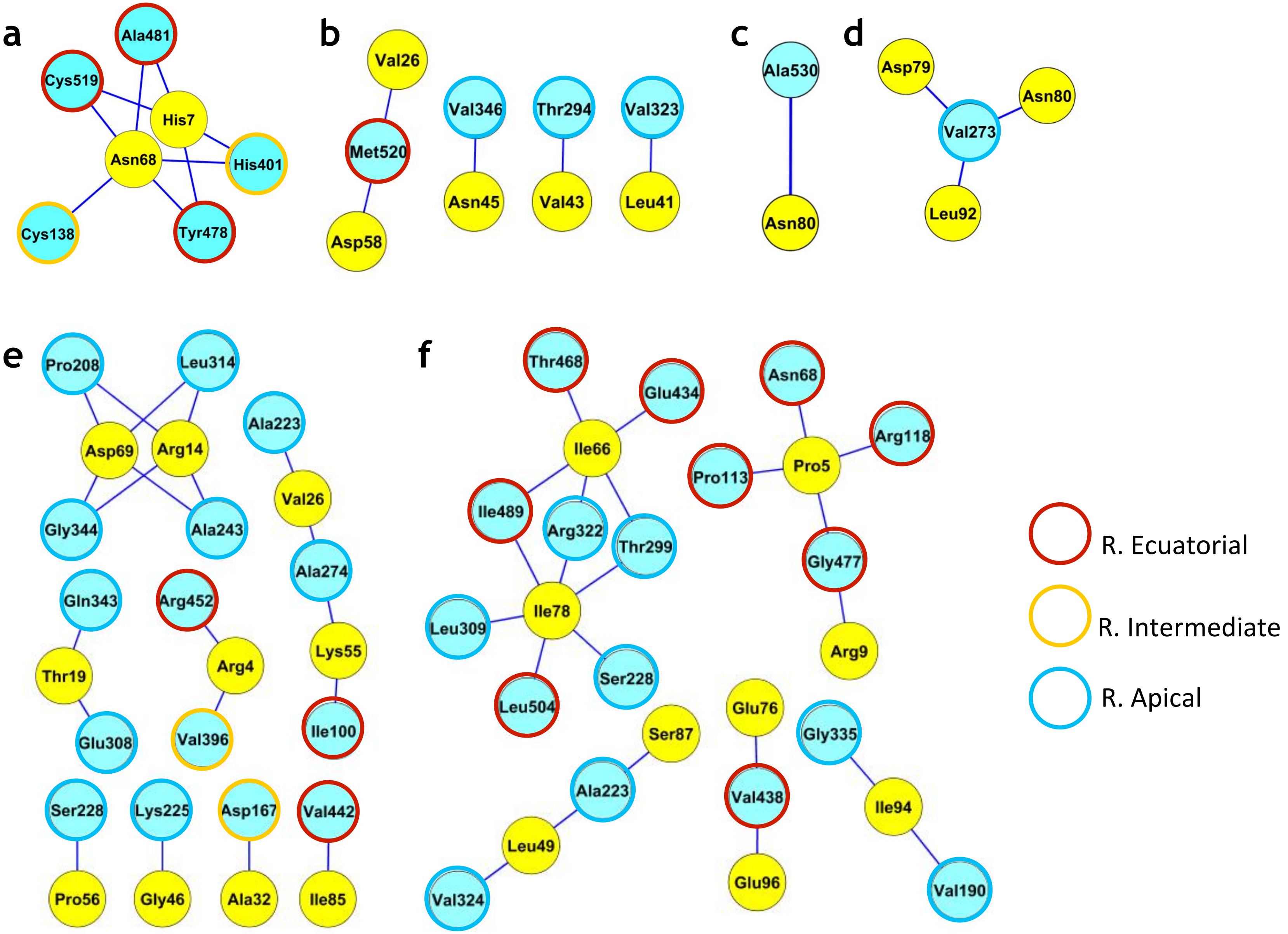
a
Ala481
Cys519
His7
Asn68
His401
Cys138
Tyr478
b
Val26
Met520
Asp58
Val346
Asn45
Thr294
Val43
Val323
Leu41
c
Ala530
Asn80
d
Asp79
Asn80
Val273
Leu92
e
Pro208
Leu314
Asp69
Arg14
Gly344
Ala243
Ala223
Val26
Ala274
Lys55
Ile100
Gln343
Thr19
Glu308
Arg452
Arg4
Val396
Ser228
Pro56
Lys225
Gly46
Asp167
Ala32
Val442
Ile85
f
Thr468
Glu434
Ile66
Ile489
Arg322
Thr299
Ile78
Leu309
Leu504
Ser228
Asn68
Arg118
Pro113
Pro5
Gly477
Arg9
Ser87
Ala223
Leu49
Val324
Glu76
Val438
Glu96
Gly335
Ile94
Val190
R. Ecuatorial
R. Intermediate
R. Apical
